# Functional Consequences of Shifting Transcript Boundaries in Glucose Starvation

**DOI:** 10.1080/10985549.2023.2270406

**Published:** 2023-11-17

**Authors:** Lan Anh Catherine Nguyen, Masaru Mori, Yuji Yasuda, Josephine Galipon

**Affiliations:** a Institute for Advanced Biosciences, Keio University, Yamagata, Tsuruoka, Japan; b Systems Biology Program, Graduate School of Media and Governance, Keio University, Kanagawa, Fujisawa, Japan; c Institute of Innovation for Future Society, Nagoya University, Aichi, Nagoya, Japan; d Faculty of Environment and Information Studies, Keio University, Kanagawa, Fujisawa, Japan; e Graduate School of Science and Engineering, Yamagata University, Yamagata, Yonezawa, Japan

**Keywords:** fission yeast, glucose starvation, transcriptome, direct RNA sequencing, transcript isoforms

## Abstract

Glucose is a major source of carbon and essential for the survival of many organisms, ranging from yeast to human. A sudden 60-fold reduction of glucose in exponentially growing fission yeast induces transcriptome-wide changes in gene expression. This regulation is multilayered, and the boundaries of transcripts are known to vary, with functional consequences at the protein level. By combining direct RNA sequencing with 5'-CAGE and short-read sequencing, we accurately defined the 5'- and 3'-ends of transcripts that are both poly(A) tailed and 5'-capped in glucose starvation, followed by proteome analysis. Our results confirm previous experimentally validated loci with alternative isoforms and reveal several transcriptome-wide patterns. First, we show that sense-antisense gene pairs are more strongly anticorrelated when a time lag is taken into account. Second, we show that the glucose starvation response initially elicits a shortening of 3'-UTRs and poly(A) tails, followed by a shortening of the 5'-UTRs at later time points. These result in domain gains and losses in proteins involved in the stress response. Finally, the relatively poor overlap both between differentially expressed genes (DEGs), differential transcript usage events (DTUs), and differentially detected proteins (DDPs) highlight the need for further study on post-transcriptional regulation mechanisms in glucose starvation.

## INTRODUCTION

Glucose is a major source of carbon used by many organisms to generate energy for cell multiplication and survival. Nevertheless, previous studies have linked an excess of glucose with metabolic disorders such as inflammation or diabetes that might induce shorter life span and promote the multiplication of tumoral cells.^1^^,^^2^ In contrast, glucose restriction has been associated with several health advantages including an increased life span in most model organisms including fission and budding yeast,^3^^,^^4^ worm,^5^ mouse and fly.^6^^,^^7^ Most recently, direct RNA sequencing (DRS) in *Schizosaccharomyces pombe* revealed alternative splicing events,^8^ confirming the fission yeast as a model of interest for the study of post-transcriptional regulation.^9^ Transcriptional shifts in stress responses such as osmotic stress, oxidative stress, and heat shock have been studied at the transcriptional level,^10^ but the response to glucose starvation remains relatively understudied. When exposed to glucose-deprived conditions, fission yeast switches from the glucose-consuming glycolysis pathway to the glucose-producing gluconeogenesis pathway.^11^ In particular, while previous studies have investigated gene expression patterns in fission yeast exposed to glucose starvation,^12–15^ the extent to which the boundaries of transcripts shift during the response is poorly understood. The 5′- and 3′-ends of transcripts define the length and therefore the sequence composition of 5′- and 3′-untranslated regions (UTRs) with implications on RNA stability and coding potential.^16^^,^^17^

This study investigates the changes in transcriptional start and end sites in the fission yeast *S. pombe* 972h- during glucose starvation to get better mechanistic insight on the transcriptional dynamics. When exposed to glucose starvation stress, the fission yeast undergoes a stress response that manifests itself at several levels of gene regulation. Previous studies have shown that TORC2 (target of rapamycin complex 2),^18^ and cAMP (cyclic AMP) signaling pathways are activated under limited glucose availability. While the former is essential for cell proliferation under glucose-limited conditions, the latter activates the protein kinase A (PKA) for inhibiting glucose starvation-induced processes, including the transcription of the *fbp1* gene that codes for fructose-1,6-biphosphatase, one of the key enzymes in the gluconeogenesis pathway.^19^ Upon glucose starvation, same-strand transcripts overlapping the entire *fbp1*^+^ locus are transcribed starting from the region upstream from the promoter, but do not produce any protein.^12^ These transcripts were subsequently defined as metabolic long noncoding RNA (mlonRNA)^13^ and were shown to participate in the full activation of *fbp1* mRNA transcription by directly recruiting transcription factors.^20^ These transcripts, as well as their antisense, harbor both poly(A) tail and 5′-cap. They are also bound by multiple ribosomes in the cytoplasm despite being noncoding, suggesting a role of RNA surveillance pathways in RNA selection during stress.^13^^,^^15^ Some of these transcripts may be seen as isoforms with an alternative transcription start site (aTSS), and may be examples of what is now known as differential transcript usage (DTU).^21^ Short-read sequencing technology has a hard time identifying these events with precision, as it is based on RNA fragmentation.^14^

Moreover, most of the data in the database of reference for the fission yeast, PomBase,^22^ still relies on short-read sequencing. Therefore, these alternative transcripts, although partially described in the literature, have yet to be referenced. With long-read sequencing, such as DRS, poly(A) tailed transcripts may be sequenced from end to end, partly overcoming this problem. The principle of this method is the native RNA going through a protein nanopore with an applied voltage. Nucleotides passing sequentially through the pore induce changes in the ionic current. Using a neural network trained on known sequences, it is possible, in theory, to precisely define the sequence of the RNA from the 3′-end to the 5′-end including the poly(A) tail length. This is important as 5′- and 3′-UTRs embed multiple regulatory units such as secondary structures,^23^^,^^24^ enriched motifs, RNA binding proteins binding sites, and small open reading frames (ORFs) in human, called upstream ORFs (uORFs) for 5′-UTRs and downstream ORFs for 3′-UTRs (dORFs), and potentially code for small peptides with functional sequences.^25–27^ In other words, there is a need to use new technologies to precisely define the 5′- and 3′-boundaries of transcripts expressed by the fission yeast, and especially during glucose starvation to better understand how these noncoding regions participate in gene regulation.

Although DRS is suitable for identifying transcript isoforms along with the position of transcription termination and the length of the poly(A) tail, the accuracy of base calling remains the lowest among existing methods. Precise mapping of the 5′-ends of transcripts is relatively more challenging due to premature runoff of some transcripts prior to reaching the 5′-end.^28^ In addition, the last 10 to 15 nucleotides at the 5′-end cannot be read.^29^ To work around these limitations, we sequenced the whole transcriptome with shotgun cDNA sequencing (formerly known as RNA-Seq) and the 5′-ends of transcripts using cap analysis of gene expression sequencing (CAGE-sequencing).^30^ By leveraging the combined advantages of the above three sequencing methods, we reannotated the fission yeast transcriptome in response to glucose starvation with unprecedented accuracy. This revealed two poorly overlapping sets of differentially regulated transcripts: differentially expressed genes (DEGs), and transcripts with shifted boundaries or DTUs. Considering DEG-antisense pairs, 25.1% and 31.9% were positively and negatively correlated with their antisense expression, respectively. In addition, we show 3′-UTRs and poly(A) tails shorten early in glucose starvation, followed by a shortening of the 5′-UTRs with functional consequences on protein domains. All in all, this study constitutes the first example of mapping the precise 5′- and 3′-boundaries of transcripts that are both capped and polyadenylated in glucose starvation.

## RESULTS

### Long-read direct RNA sequencing combined with short-read cDNA sequencing and 5′-CAGE allows reannotation of the transcriptome of fission yeast during glucose starvation

**FIG 1:**
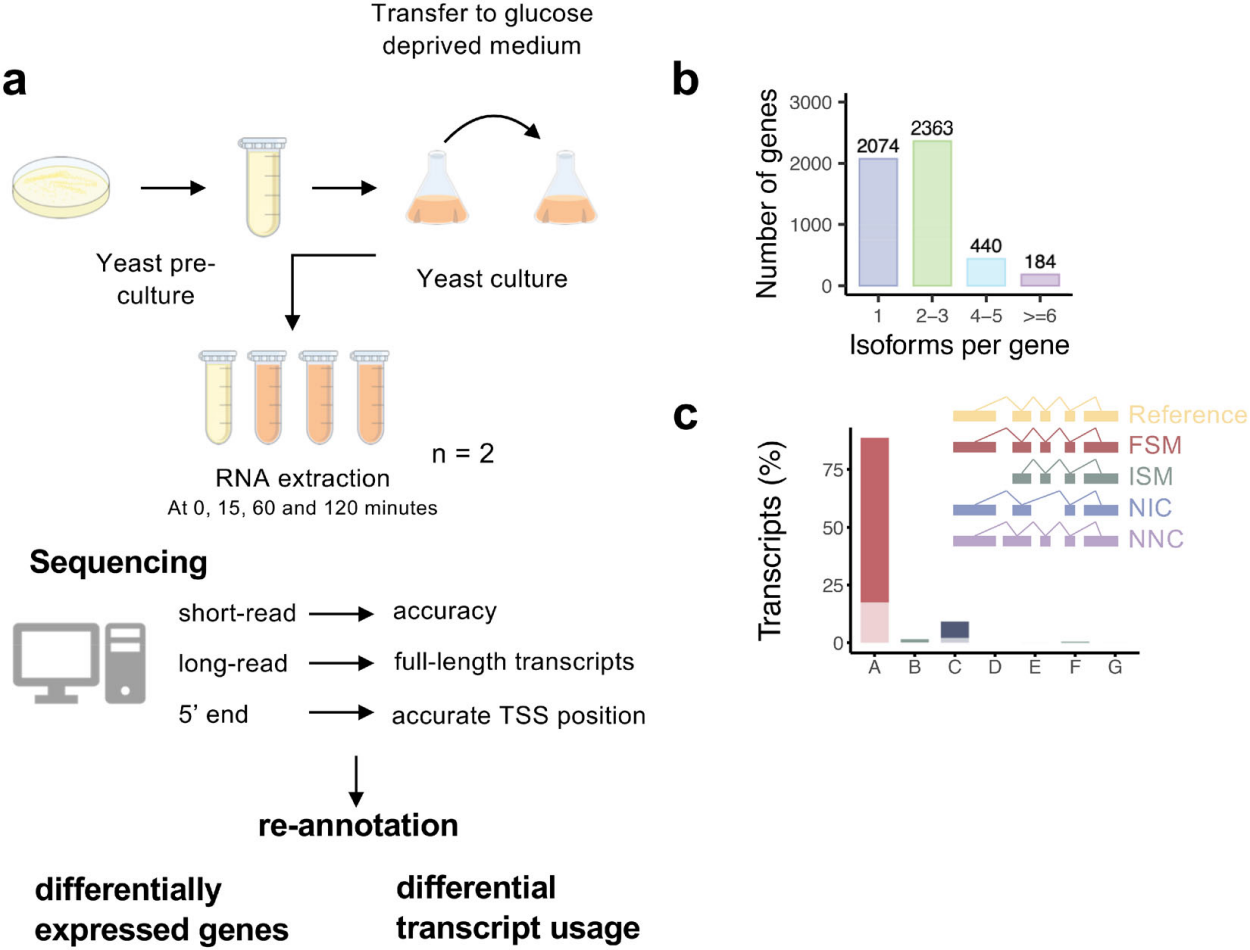
Overview of the study and different types of transcripts after reannotation. (A) Workflow of the study of fission yeast exposed to glucose starvation with the different sequencing technologies implemented (B) Number of transcript isoforms found per gene (C) Various types of transcripts as classified by SQANTI3 workflow with the percentage of the transcripts belonging to each category A – FSM, B – ISM, C – NIC, D – genic genomic, E – antisense, F – fusion, G – intergenic.

As shown in Fig. 1a, fission yeast is exposed to glucose starvation for 120 min: after growing in glucose rich medium (6% glucose), the cells are transferred to a medium with only 0.1% glucose during exponential growth, inducing a stress response that is similar to glucose starvation but which does not induce cell death.^31^ Total RNA is extracted at four different time points: 0 (rich), 15 (early), 60 (mid), and 120 (late) min. In order to increase the accuracy of 5′- and 3′-end identification, the transcriptome was sequenced using three methods: (a) long read direct RNA sequencing and (b) shotgun cDNA sequencing (RNA-seq) on poly(A)-enriched RNA, and 5′-CAGE. Using FLAIR and SQANTI3 analysis pipelines to combine these datasets, the fission yeast transcriptome was reannotated taking into account all transcript isoforms during glucose starvation and compared with the reference transcriptome annotation available in PomBase.

In this study, the annotation produced by FLAIR/SQANTI3 will be referred as ‘SQANTI’ while the annotation produced by merging the existing PomBase annotation with our newly generated SQANTI annotation will be called “PomBasePlus”.

The newly identified transcripts are characterized in Fig. 1b–d. As shown on Fig. 1b, most of the genes had between 1 and 2–3 isoforms (41.0% and 46.7% respectively) and a small portion of genes had more than four isoforms (Supplementary Table 1) (12.3% (624/5061)). When compared to the reference, SQANTI3 categorizes the transcripts into various categories: full splice match (FSM), incomplete splice match (ISM), novel in catalog (NIC), and not novel in catalog (NNC). ISM are isoforms that “have fewer 5′ exons than the reference, but each internal junction agrees. The exact 5′ start and 3′ end can differ by any amount”. NIC transcripts are defined as an isoform that “does not have a FSM or ISM, but is using a combination of known donor/acceptor sites” (citation from the SQANTI user manual).^32^^,^^33^ As observed on Fig. 1c, most of the transcripts in the annotation produced by the pipelines are FSM (9,839 (88.7%) in brown), 1,020 (9.2%) are NIC, and 175 (1.6%) are ISM.

**FIG 2:**
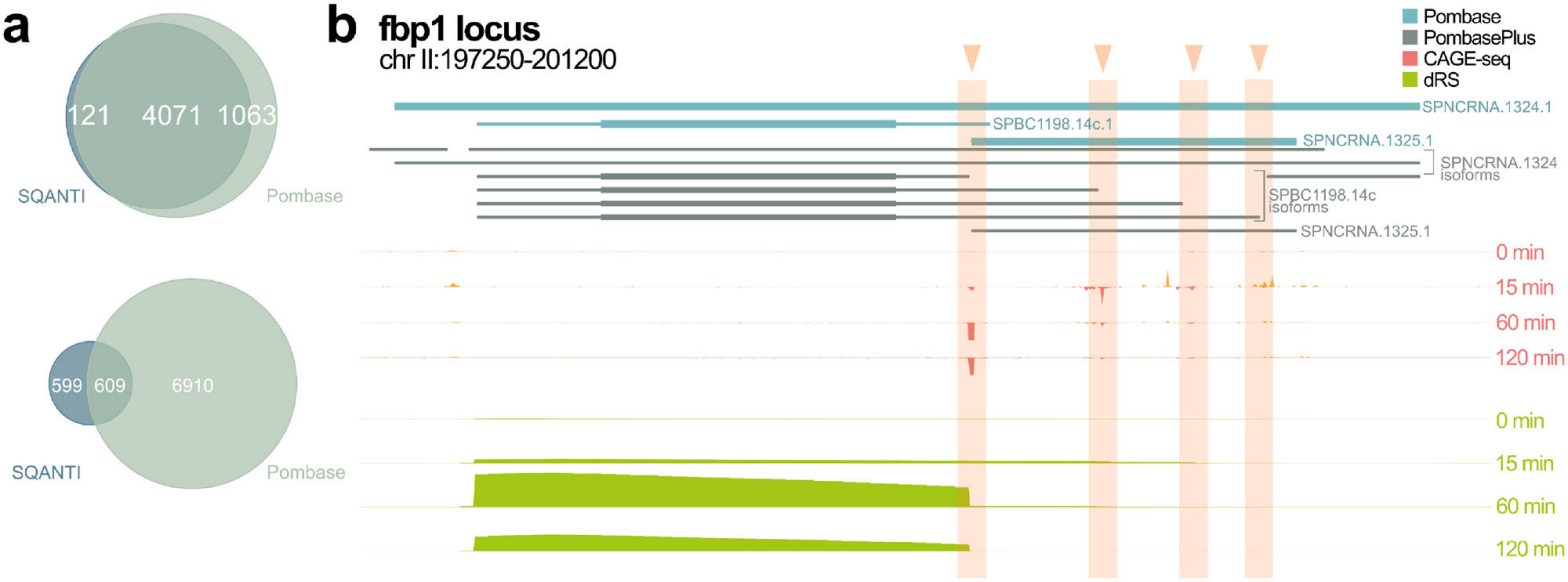
Comparison of transcriptome reannotation with the PomBase database. (A) Overlap of coding genes between SQANTI and PomBase and overlap of non-coding genes between SQANTI and PomBase (C) PomBase and PomBasePlus annotation and next generation sequencing (NGS) tracks at *fbp1*^+^ locus. For CAGE-seq peaks, orange represent strand positive tracks while red represent strand negative tracks. For DRS, only strand negative tracks are shown.

We also defined the overlap between coding and noncoding transcripts found in both SQANTI and PomBase’s annotation. Moreover, 38 genes detected as fusion genes were considered artefacts and removed before further analysis. The coding potential of genes was assessed by GeneMark from the SQANTI3 workflow. Coding genes, which represent 77.5% of all the genes, found in both datasets. A small portion of the coding genes are newly found by SQANTI (121, 2.30%). After mapping these genes, we found that they map to both a coding and a noncoding gene which is annotated as “predicted untranslated region (UTR)”. However, the overlap was smaller for noncoding genes: only 627 (7.67%) are found in both SQANTI and PomBase. Some of the genes not found in the overlap are genes that were erroneously classified as noncoding by GeneMark based on the small size of the ORF, despite being categorized as coding in PomBase and experimentally validated. Among these genes, there are also unlisted antisense genes, which were (Supplementary Table 1) opposite of *alp31^+^* (SPAC8E11.07c), *mam2^+^* (SPAC11H11.04) and *erg4^+^* (SPAC20G4.07c). Genes that were only present in PomBase’s annotation include genes that were described in previous literature and may also include genes giving birth to noncapped and/or nonpolyadenylated transcripts. Hence, Fig. 2a shows that our reannotation obtained with the FLAIR/SQANTI3 pipelines is for the most part consistent with the existing PomBase annotation in terms of gene content, with a number of novel coding and noncoding transcripts that correspond to 7.6% and 2.4% of their known annotated transcripts respectively. The list of all transcripts and their respective genes are found in Supplementary Table 2. The gene content is consistent with the previous annotation, but our pipeline’s purpose is to provide new insights on the precise 5′- and 3′-ends of transcripts, the results of which are described in the following sections.

### Differential gene expression shows an upregulation in carbon metabolism associated with a downregulation in ribosome biogenesis

In response to glucose starvation, the fission yeast shifts its metabolism by inducing or repressing the gene expression.

**FIG 3:**
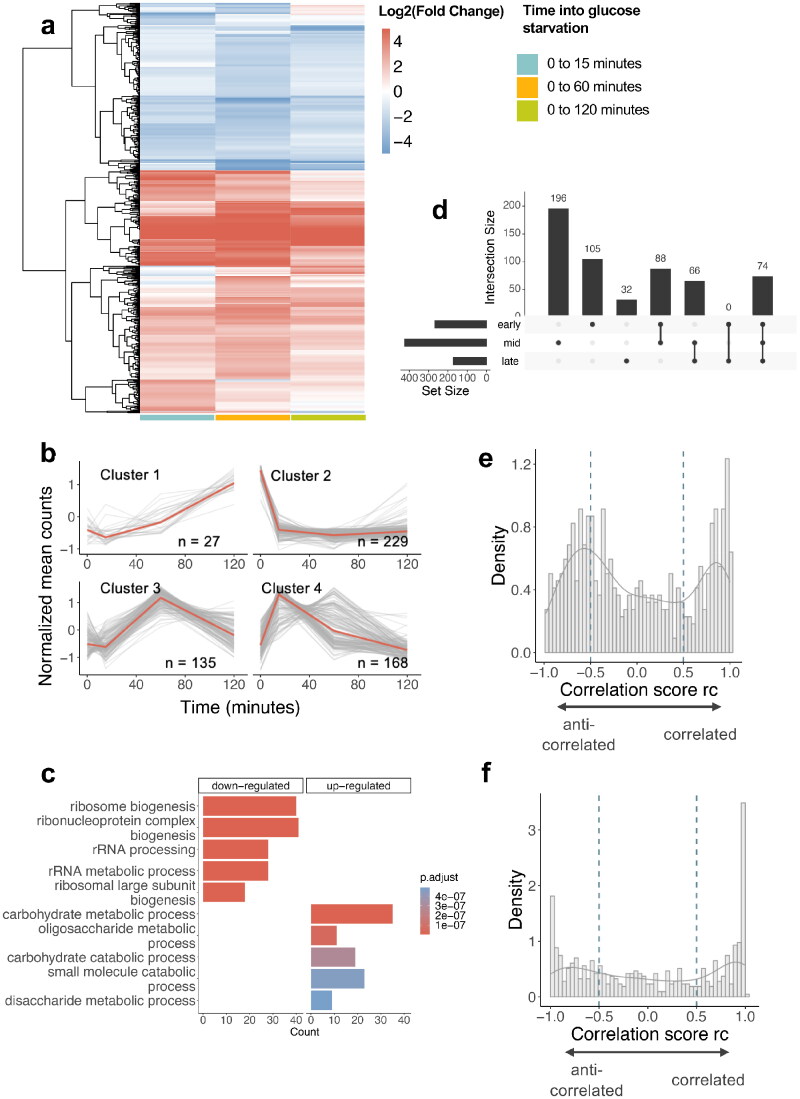
Transcriptomics during glucose starvation. (A) Heatmap of gene expression of the differentially expressed genes (DEGs) during glucose starvation compared to glucose rich condition (cutoffs: |log_2_(FC)| > 2, *p*-adj < 0.05, n = 2). (B) KEGG enrichment pathway for downregulated and upregulated DEGs ranked by *p*-adj (C) Upset plot showing the overlaps between the DEGs found at each stage into glucose starvation (early: 15 min, mid: 60 min, late: 120 min). Supplementary Table 4 shows the different genes in each category, going from left to right. (D) Clustering of gene expression of the DEGs during glucose starvation (E) Correlation score r_c_ distribution for pairs of DEG with their antisense (with no lag) (F) Correlation score r_c_ distribution for pairs of DEG with their antisense (with lag).

Fig. 3a shows the evolution of gene expression patterns during glucose starvation that were observed among 561 significantly differentially expressed genes (DEGs) which were defined as genes with a significantly different expression level at least one time point compared to the glucose-rich condition (0 min) (|log2(Fold Change)| ≥ 2, *P* value ≤ 0.05, Wald test). These DEGs represent 5.7% of the gene set in our new PomBasePlus annotation (9,684 genes). More DEGs are observed at 60 min (424 (upregulated:downregulated) (251:173) genes compared to 265 (188:77) and 172 (123:49) genes for 15 and 120 min, respectively), suggesting that although some genes are induced or repressed earlier on, the stress response induced the biggest change in terms of number of DEGs at 60 min into glucose starvation.

Hierarchical clustering on the DEG’s expression during glucose starvation revealed four clusters: cluster 1 (permanent induction) with 27 genes, cluster 2 (permanent repression) with 229, cluster 3 (mid-transient repression) with 135 and cluster 4 (early transient induction) with 168 genes (Fig. 3b and Supplementary Table 3). KEGG pathway enrichment analysis (Fig. 3c) on both upregulated and downregulated genes at all time points (false discovery rate, FDR, < 0.05) showed that upregulated genes tend to be involved in carbohydrate metabolic process and oxidoreductase activity. In contrast, downregulated genes are involved in ribosome biogenesis, rRNA processing and ribonucleoprotein complex biogenesis, consistent with previously observed transient global translation inhibition observed in glucose starvation.^13^^,^^34^ It is likely that the fission yeast reallocates some of the resources that were spent on ribosome biogenesis for use in carbon metabolism to ensure survival during the shift. The KEGG pathway enrichment changes dynamically over the course of the response, consistent with previously described resuming of translation and cell division at 120 min (Supplementary Table 2).

When comparing the overlap of DEGs between the different stages into glucose starvation (early, mid, and late) (Fig. 3d and Supplementary Table 4), early and mid-stages overlap by 88 genes while mid and late overlap by 66 genes. Over the course of the glucose starvation response, 74 genes are constantly differentially expressed compared to glucose-rich. In contrast, a higher number of genes were differentially expressed at only one stage (105, 196, and 32 genes respectively for 15, 60, and 120 min), in other words showing a transient expression change. Supplementary Fig. 1 also shows that the global correlation coefficient between RNA expression at 15 and 60 min (*r*^2^ = 0.85) is lower than the one between 60 and 120 min (*r*^2^ = 0.94), suggesting that although the number of DEGs itself was the highest at 60 min, the overall amplitude of the change was biggest at 15 min.

### A subset of differential expressed genes is associated with antisense transcription on the opposite strand

Previous studies on the *fbp1^+^* gene showed that its expression in glucose starvation is anticorrelated with the expression of antisense RNA on the opposite strand. Our new annotation leveraging full-length RNA sequencing allowed us to identify all four previously sense-strand overlapping transcripts as well as three antisense RNA isoforms on the opposite strand, one of which is novel and overlapping with the 3′-end of sense transcript but not with the ORF.^14^ We found 538 pairs of DEG-antisense, with some DEGs associated with multiple antisense transcripts based on the annotation file. Among these, 469 unique DEGs out of the total 561 were associated with at least one antisense (83.6%). This was not so different from the genome-wide value: 8,378 expressed genes out of 9,716 which had an antisense (86.2%), suggesting that DEGs are not particularly enriched in antisense annotations. To better understand the mechanisms, we investigated the potential co-expression between these pairs.

To determine whether a pair of sense-antisense is correlated or anticorrelated, a compositional correlation score r_c_ was calculated (for details, cf Material and Methods section), with r_c_ < –0.5 indicates anticorrelation and r_c_ > 0.5 correlation. Fig. 3e shows the distribution of the r_c_ for these pairs. The number of DEGs that are either positively or negatively correlated with their antisense are roughly equal in size (168 and 160, respectively) when no time lag is considered.

Next, we hypothesized that the antisense gene transcription may temporally precede the sense transcription, as suggested by previous studies showing a regulatory effect of the antisense on the remodeling of the chromatin structure in *cis*.^35,36^ However, past studies in fission yeast do not take into a possible delayed effect when studying correlation. To investigate this, the r_c_ with the DEG’s expression at time points 15, 60, and 120 min was calculated as an input for a scalar vector A, and at time points 0, 15, and 60 min for the associated antisense gene as an input for a second scalar vector B. The distribution of the r_c_ for the same pairs at lagged time points in the glucose starvation is shown on Fig. 3f.

Table 1 shows that when considering a time lag, significantly more strongly anticorrelated gene pairs (60 genes with r_c_ ≤ –0.9) were found than when comparing simultaneous time points (19 genes with r_c_ ≤ –0.9 for no lag). This suggests that for anticorrelated sense-antisense pairs, expression of the antisense does indeed tend to precede the sense’s expression. Interestingly, a population of positively correlated sense-antisense pairs were detected in roughly equivalent numbers, suggesting that some opposite-strand pairs of genes may operate using a different mechanism. Correlation scores r_c_ for each pair of DEG-antisense, both non-lag and lag, are presented in Supplementary Table 5 along with their expression levels

**TABLE 1:** Number of gene pairs with an absolute correlation score more than 0.5 and 0.9

no lag	≤ –0.5	160
	≥ 0.5	168
lag	≤ –0.5	179
	≥ 0.5	141
no lag	≤ –0.9	19
	≥ 0.9	65
lag	≤ –0.9	60
	≥ 0.9	69

When testing for antisense transcription preceding that of sense transcription, 25.1% of all DEG-antisense pairs are positively correlated, and 31.9% are anticorrelated. The former is likely to operate by a different mechanism than the latter.

### Differential transcript usage induces protein domain gain and loss during glucose starvation

In addition to differential gene expression, it has been previously reported that fission yeast uses alternative transcription start sites (aTSS) in other stress responses (nitrogen depletion, heat shock or oxidative stress).^10^ Our analysis reveals 322 differential transcript usage events (DTUs), defined by alternative 5′- and 3′-ends but not necessarily any change in gene expression levels.

**FIG 4:**
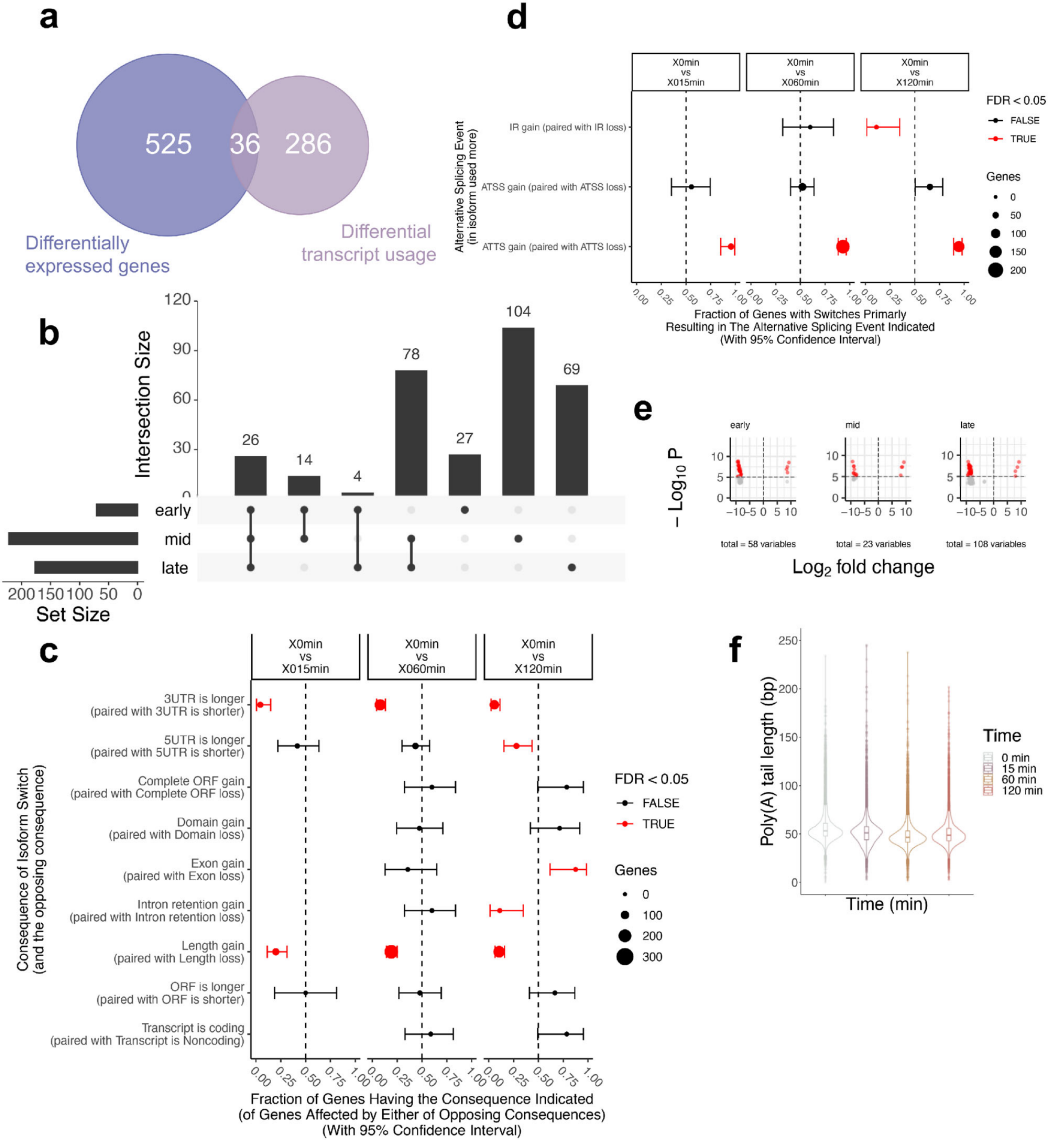
Differential transcript usage during glucose starvation. (A) Venn diagram showing the overlap between DEG and DTU. (B) Upset plot showing the overlaps between the DTUs found at each stage into glucose starvation (early: 15 min, mid: 60 min, late: 120 min). Supplementary Table 7 shows the different genes in each category, going from left to right. (C) Fraction of genes having the consequence indicated when compared to glucose rich condition. (D) Fraction of genes having the consequence indicated when compared to glucose rich condition. (E) Volcano plot with the log_2_ fold change of the poly(A) tail length as x-axis with -log_10_ *P* value. Genes with a significant change (*P* value ≤ 0.05) in their poly(A) tail length are highlighted in red, while genes with no significant change are highlighted in grey. (F) Violin plots of the distribution the poly(A) tail lengths at each time into glucose starvation for each gene.

When comparing the overlap between all DEGs (561) with the DTUs identified here, an overlap of 36 genes was observed (Fig. 4a). The relatively low overlap between these two sets of genes suggests that that fission yeast regulates its metabolism in a bimodal manner (Supplementary Table 6 shows the DEG and DTUs with the overlap). Genes with isoform switches have various consequences, which are investigated below.

Like for the DEGs, the overlap between the DTUs found in the early and late stages is minimal (four genes) compared to the overlap between the other time points: the early-mid and mid-late overlapped by 14 and 78 genes, respectively. Twenty-six genes are consistently identified as a DTU compared to the glucose rich condition (Supplementary Table 7). Similarly, distinct sets of genes are DTUs at each time point during glucose starvation, again consistent with dynamic regulation within the first two hours of glucose starvation.

Associated with the various alternative isoform switch events, changes in functional consequences happen. Transcripts are significantly shorter with a length loss in 3′-UTRs at all time points during the stress (for 44, 151, and 117 genes at early, mid and late stages respectively) and in 5′-UTRs (for 32 genes) later into glucose starvation (120 min). One advantage of DRS is the possibility to assess the length of the poly(A) tail of each transcript. A significant shortening of the poly(A) tail length is observed as well, though with some genes had a longer poly(A) tail (SPCC1919.12c, SPNCRNA.1223, SPNCRNA.4537, SPNCRNA.964 at all time points, and SPNCRNA.3213 only at 15 min) (Fig. 4e and f). We used the Wilcoxon signed-rank test to compare the poly(A) tail length at each time point with 0 min as described in the Materials and Methods section. The *P* values are extremely small for all time points compared to glucose-rich, indicating that the poly(A) tail length is significantly smaller at each time point in glucose starvation (Table 2). Overall, there is a global shortening of the poly(A) tail length. We also noticed that genes with a shorter 3′-UTR and genes with a shorter poly(A) tail did not overlap.

**TABLE 2:** Related *t* test results comparing poly(A) tail lengths at different time points during glucose starvation

	Statistic value	*P-*value
15 min vs 0 min	15257928.0	1.65E-224
60 min vs 0 min	9782070.0	0.0
120 min vs 0 min	11384537.0	0.0

Among the 9,888 genes that have a poly(A) tail at any time point, 159 genes have a poly(A) tail with a significant change in length. Interestingly, 89.3% of these genes were noncoding (142/159) with one among the coding genes being dubious (Supplementary Table 8). The changes in poly(A) tail length when comparing 60 min to glucose rich are less prominent compared to the changes at 15 and 120 min.

Associated with shorter 5′- and 3′-UTRs, domains gains and losses are also discovered for a small set of genes (15 and 12 domains respectively). The gained and lost domains detected at each time point are described in Supplementary Table 9. These domains were predicted computationally by Pfam and we quantified the peptides from our proteomics data (cf. Materials and Methods) at the domain level to validate or the gain and/or loss as presented in Supplementary Table 10. Domains that were gained during glucose starvation by isoform switching include domains involved in the transcriptional activity of RNA polymerase II and transcription elongation regulation, vegetative growth, spore formation and mitophagy. Domain losses were observed on proteins involved in sugar transport and ribosome subunits such as ribosomal proteins L29, L35ae, and S17, which is consistent with the downregulation in ribosome biogenesis described in the previous section.

### Upregulated differential detected proteins are also involved in carbon metabolism and downregulated ones are involved in endocytosis, promoting the fission yeast cells’ survival

**FIG 5:**
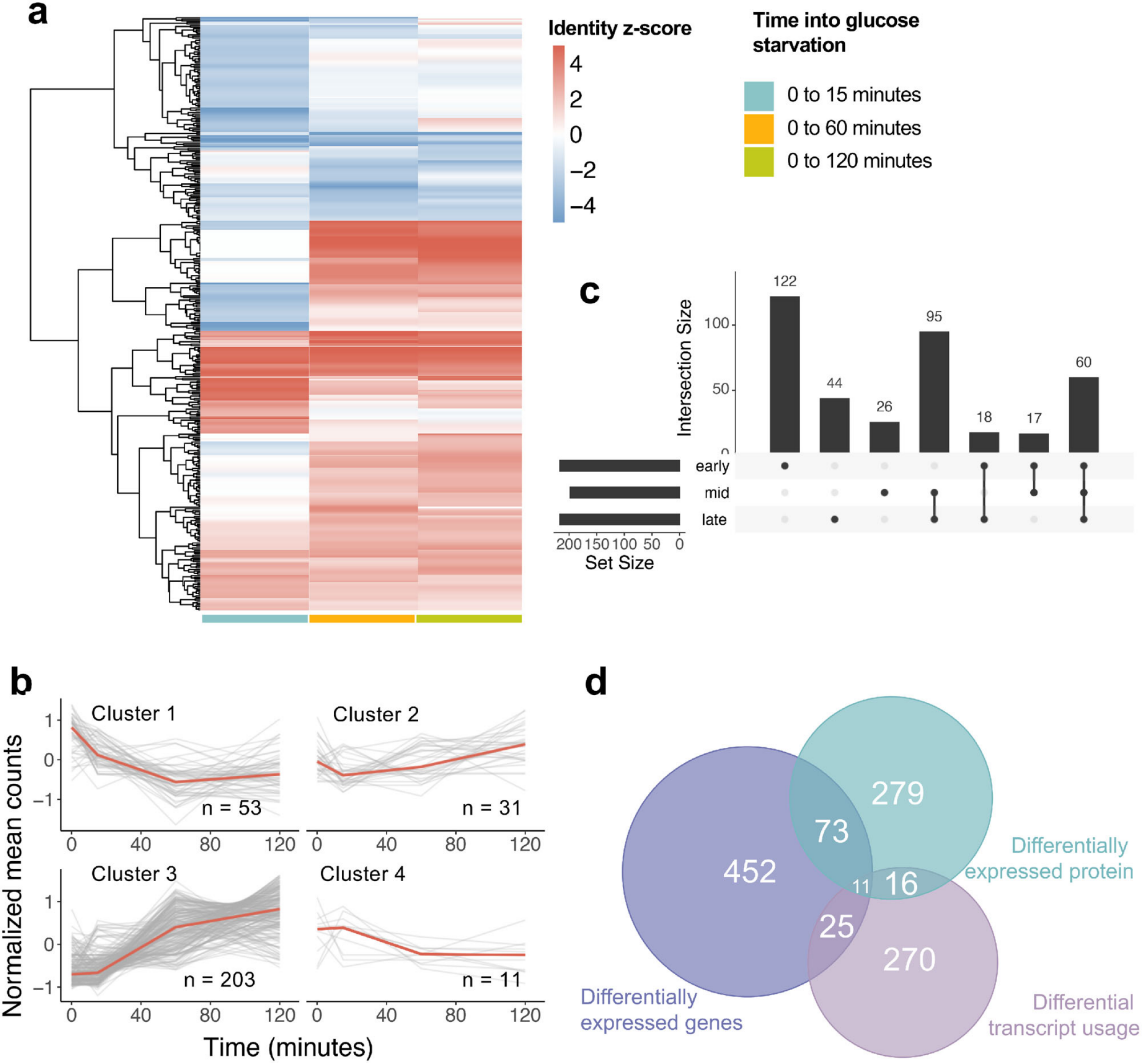
Proteomics during glucose starvation. (A) Heatmap of protein detection using z-score of differentially detected proteins (DDPs) during glucose starvation compared to glucose rich condition (cutoffs: |z-score| > 2, n = 4). (B) Clustering of the protein intensity of differentially expressed proteins during glucose starvation. (C) Upset plot showing the overlaps between the DDPs found at each stage into glucose starvation (early: 15 min, mid: 60 min, late: 120 min). Supplementary Table 12 shows the different genes in each category, going from left to right. (D) Venn diagram showing the overlaps between DEGs, DTUs and DDPs.

The effect of glucose starvation on the proteome landscape of the fission yeast was studied at the same four time points. In contrast with the results of transcriptome analysis, where induced transcripts tend to be expressed at a basal level in glucose rich, many proteins were not detected at all in glucose rich but specifically produced later in the stress response. The z-score calculated by MS-DAP for differential detection was the basis for our analysis. Fig. 5a shows differential z-score patterns during glucose starvation for 379 proteins with a |z-score| > 2 at any time point into the stress, defined as differentially detected proteins (DDPs). This represents approximately 7.4% of all the proteins listed in UniProt database for the fission yeast (out of 4,021 proteins detected). Interestingly, compared to RNA expression, we see that there are more DDPs at early stages compared to mid stages (217 (upregulated:downregulated) (95:122) proteins at early step, 198 (145:53) at mid and 217 (177:40) at later stages). While there are more DDPs at 15 min, 56.2% (122/217) are downregulated, later stages into the stress have a reversed ratio of upregulated to downregulated proteins, with upregulated ones being more dominant.

KEGG pathway enrichment analysis on upregulated proteins shows an enrichment in proteins involved in the TCA cycle, 2-oxocarboxylic acid metabolism, biosynthesis of amino acids and carbon metabolism, while the downregulated proteins are involved in endocytosis, purine metabolism, nucleocytoplasmic transport and spliceosome. Similarly, we clustered the DDPs’ intensity during glucose starvation into four clusters, cluster 1 (permanent repression) with 53 proteins, cluster 2 (early transient repression) with 31, cluster 3 (permanent induction) with 203 and cluster 4 (early transient repression) with 11 proteins (Fig. 5b). The list of proteins in each cluster is presented in Supplementary Table 11. Cluster 2 is significantly enriched in protein K11-linked ubiquitination, positive regulation of autophagy, anaphase-promoting complex-dependent catabolic process and Golgi to plasma membrane transport for biological processes.

**FIG 6:**
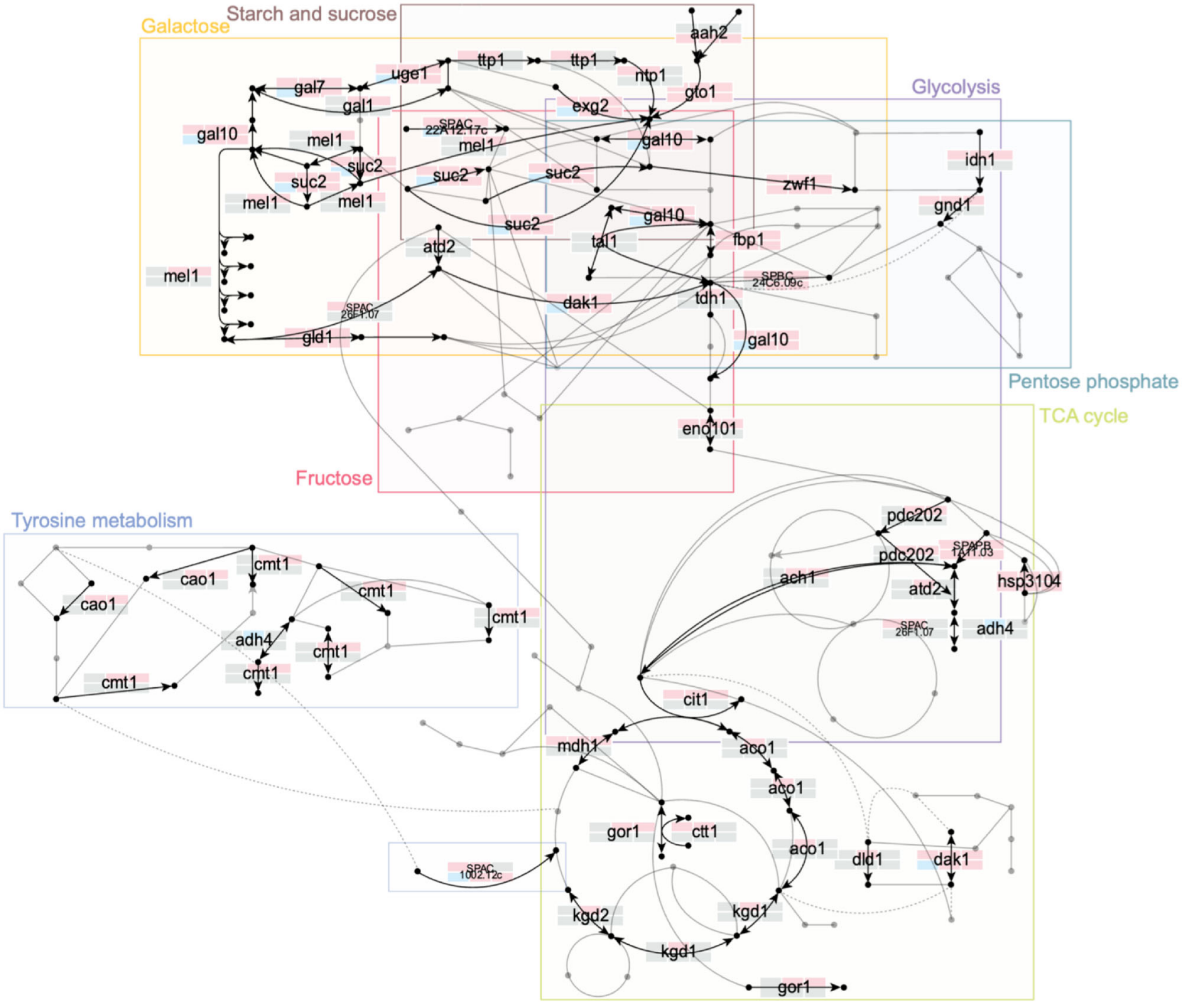
Integration of transcriptome and proteome analysis with metabolic maps. Each arrow corresponds to a reaction. Each gene producing the protein associated is written on the figure if expressed at any time point or any transcript or protein level. For each gene, their expression during glucose starvation is shown at each time point (15, 60, and 120 min compared to 0 min). The first row is the expression at the gene level while the second row is the expression at the protein level. If the expression is upregulated compared to 0 min, the rectangle is red. If it is downregulated, it is blue. If it is grey, it is not expressed at this specific time point.

We also compared the overlap of DDPs at different stages into glucose starvation (Fig. 5c and Supplementary Table 12). As observed previously, the mid and late stages have the biggest overlap (95 proteins), while early/mid and early/late have a smaller one (17 and 18 proteins, respectively). This suggests that the proteome changes most dynamically in the early stage (15 min). Likewise, Fig. 5d shows the overlap between DEGs, DTUs and DDPs at all time points into glucose starvation. The overlap between DEGs and DDPs is 15.0% of all DDPs and that between DTUs and DDPs is 8.4% of all DTUs, confirming previous results showing that the correlation between gene expression and protein expression is low (Supplementary Fig. 3). Hence, both DEGs and DTUs are partially involved in the changes in the translational response. DEGs only are enriched in proline transmembrane transport, proline import across plasma membrane, trehalose metabolic process, cellular response to heat and disaccharide metabolic process. For the DTU, these are enriched in membrane protein complex, Golgi apparatus, organelle component, organelle membrane and endomembrane system when doing gene ontology analysis on cellular component. Fig. 6 summarizes the major metabolic pathways with different genes involved and their gene and protein expression during glucose starvation. Most DEGs and DDPs are involved in starch and sucrose, galactose, glycolysis, fructose, pentose phosphate, TCA cycle and tyrosine metabolism, with both DEGs and DDPs involved in starch and sucrose and galactose metabolisms upregulated during glucose starvation, with a lag for DDPs where they are downregulated first.

## DISCUSSION

In the past decades, new sequencing technologies have emerged and improved allowing the discovery of new mechanisms of gene regulation. In this study we focus on identifying differential 5′- and 3′- transcripts in glucose starvation in fission yeast.

As a result, differentially expressed genes during glucose starvation were classified into four clusters depending on the timing of their activation. The gene ontology results for these correspond well to previous results described in Oda et al.,^14^ therefore confirming that our reannotation, while providing much more detail at the transcript isoform level, is largely consistent with past literature at the gene level.

Our study, by allowing us to precisely define the boundaries of overlapping transcripts, also allowed us to identify DEG-antisense pairs at the isoform level in greater detail. These include known DEG-antisense isoforms in glucose starvation that have been previously confirmed by Northern blotting.^14^^,^^15^ As Muskovic et al.^37^ previously suggested, the transcription of regulatory lncRNAs precedes the changes in the sense gene expression, we showed that in the fission yeast, the correlation between the expression of DEGs and their antisense is more highly correlated with a time delay into glucose starvation, suggesting that the antisense genes are transcribed first. We also found that antisense transcription concerns around 86.2% of genes. Wery et al.^38^ found a similar percentage of 76.9% in fission yeast. A slight discrepancy of these percentages can be explained by updates in the PomBase database since 2018 (date of publication of Wery et al.’s study) and the fact that they are using different sequencing methods, in different growth conditions. In addition, we found two populations of pairs of antisense-sense genes, one with anticorrelated gene expression (31.9%) and the other one with correlated gene expression (25.1%). Moreover, previous studies that describe anticorrelation as their main finding base their conclusions on a global trend using Spearman rank correlation between antisense and sense expression variables and did not expose the fission yeast cells to any stress,^38,39^ explaining the difference with our results. Previous studies also show the existence of both positively and negatively correlated antisense-sense pairs,^40^ with divergent pairs of antisense-sense being anticorrelated and the convergent ones being positively correlated. While our study shows two different populations of antisense-sense pairs as Leong et al. found, we did not find a clear relationship between the divergence of these pairs and their correlation score. In conclusion, we observed both a positive and negative correlation in antisense-sense pairs’ gene expression during glucose starvation in a time lag. This aspect is relatively novel as previous studies have not considered the time lag when assessing correlation.

Following this, the precise quantification of overlapping transcripts unveils aTSS and aTTS switches happening during glucose starvation which have not been described before in the fission yeast. Globally, we observed significant shortening of the 3′-UTRs, alongside with poly(A) tail length from early stages into glucose starvation. 5′-UTRs are significantly shorter at later stages. Studies in other organisms exposed to several stresses have shown changes in 3′-UTRs and poly(A) tail lengths mainly.^41–46^ 3′-UTRs shortening and lengthening studies are still incomplete, however, studies in human and mouse cells suggest that a shortening increases mRNA stability by limiting the access to mRNA degradation mechanisms (such as ribosome binding proteins or miRNA).^41,47–49^

As opposed to genes with a shorter 3′-UTR, genes with a shorter poly(A) tail length are mainly noncoding genes. Unlike mRNAs, the presence of a poly(A) tail on noncoding RNAs is a signal for the degradation of the transcript.^50^ While past research has investigated the impact of poly(A) tail length on the mRNA, including its stability,^51^ its expression,^52^ and its translational efficiency,^53^ no previous research has been done to describe the impact of the changes in poly(A) tail length on noncoding RNA. Since mRNA with shorter poly(A) tails are targeted for degradation, we hypothesize that the shortening of the poly(A) tails may also be a signal for degradation of the noncoding RNAs during glucose starvation. Further investigations are necessary to decipher the mechanism by which these specific noncoding transcripts are targeted.

The isoform switches directly impact the length of both the ORFs and the UTRs, inducing changes in proteins domains for 32 genes and 52 different protein domains (Supplementary Table 9) involved in transcriptional activity and mitophagy and were validated by proteome analysis. Zheng et al. showed in a previous study that complete starvation of the yeast induces mitochondrial fragmentation.^54^ While previous studies have inquired into protein domain changes (gain and loss) between various organisms,^55–57^ our research is the first one to describe protein domain changes during glucose starvation induced by aTSS and aTTS.

On a translation level, as observed in previous studies in various organisms and confirmed by our results (Supplementary Fig. 3),^58–60^ correlation between gene expression and protein detection is poor. The use of different technologies and methodologies for detecting the transcripts counts and the protein abundance,^61^ on a technical level, and a time delay between mRNA and protein synthesis, with their stability and half-life being distinct, on a biological level, explain this poor correlation. Both DEGs and DTUs are partially overlapping with DDPs, implying that two different mechanisms are involved in the post-transcription response regulation. DEGs, with their changing expression directly impact changing protein abundance. DTUs, while having their boundaries changed, their expression is not, which indicates that changes in protein abundance might involve other mechanisms such as RNA half-life, protein half-life or the ribosomal activity for these transcripts. Globally, in the early stages of glucose starvation, gene ontology shows an enrichment in permeases and transporter proteins among the downregulated proteins, and in proteins involved in ubiquitination and autophagy among the upregulated. Previous studies have demonstrated autophagy events happening during glucose starvation in fission yeast.^62^ These results suggest an activation of protease activity in the early stages of glucose starvation, possibly by the recognition of ubiquitinated substrates, which are then digested into peptides.^63^ While earlier investigations also showed ubiquitination of ribosomal protein uS3 in budding yeast during rapid stress response,^64^ additional analysis involving measurements of ubiquitination levels and enzymatic activity of the proteases could be done to validate our results in glucose starvation.

In conclusion, by comprehensively reannotating the 5'- and 3'-boundaries transcript isoforms that are both 5'-capped and polyadenylated in glucose starvation using a combination of three different sequencing methods, our study unveils that both changes in gene expression (DEGs) and shifting of transcript boundaries (DTUs) contribute to the fission yeast response to glucose starvation, with functional consequences at the protein level in terms of domain gain/loss. Our data also shows that the anticorrelation observed at sense-antisense pairs tends to be stronger when a time lag is taken into consideration, and that the poly(A) tails of many noncoding RNAs are shortened in glucose starvation. On messenger RNA, 3'-UTRs shortening followed by 5'-UTRs shortening, highlight the need for in-depth follow-up studies to evaluate the impact of glucose starvation on ribosomal activity, RNA stability, and translation efficiency.

## MATERIALS AND METHODS

### Strains pre-culture and culture

20 mL of cells were harvested for time point 0 min from the yeast extract rich (YER) medium (0.5% w/v yeast extract; 6% glucose; 100 mg/L adenine) when the concentration reached 1.8–2.2 × 10^7^ cells/mL. The remaining cells were transferred to yeast extract dextrose (YED) medium (0.5% w/v yeast extract; 0.1% glucose; 3% glycerol; 100 mg/L adenine) after washing them twice with distilled water. 20 mL of the cell cultures were collected by centrifugation at different time points (15, 60, and 120 min), and rinsed once in ice-cold 1X phosphate buffered saline (PBS) (137 mM NaCl; 2.7 mM KCl; 10 mM Na_2_HPO_4_; 1.8 mM KH_2_PO_4_). After sampling, the cells were flash-frozen in liquid nitrogen and stored at –80 °C.

### RNA extraction

Frozen pellets were re-suspended in 250 µL of 65 °C sterilized bead buffer (7.5 mM NH_4_OAc; 10 mM EDTA, pH 8.0) and then transferred to 1.5 mL tubes prewarmed at 65 °C acid-washed glass beads (G8772-100 Merck) suspended in 25 µL of 10% filtered SDS and 300 µL of acid phenol chloroform pH 4.5. The tubes were vortexed for 1 min then incubated at 65 °C for 1 min. This was repeated three times. After heating the tubes at 65 °C for 10 min, and after an additional 1 min-round of vortex, the samples were centrifuged at 16,000 × *g*, 25 °C, for 15 min. The supernatant was then transferred to a new tube with an equal volume of chloroform on ice. After vortexing the tubes, the tubes were centrifuged at 16,000 × *g*, 4 °C, for 15 min. The supernatant was transferred to a new tube with 7.5 M NH_4_OAc (final concentration of 0.51 M). The tubes were then vortexed. After adding 100% cold ethanol, the tubes were put at –80 °C. The samples were then centrifuged at 20,400 × *g* at 4 °C for 30 min. After discarding the supernatant, the pellet was washed in 75% ice-cold ethanol was then added. After a final centrifugation at 20,400 × *g*, 4 °C, for 15 min, the supernatant was discarded. The pellet was dried using a vacuum-centrifuge for 5 min and re-suspended on ice with cold RNAse-free water and stored at –80 °C. RNA quality and concentration was measured using a Nanodrop spectrophotometer (ND-1000).

### Direct RNA library preparation and sequencing

RNA library preparation was done using direct RNA sequencing (DRS) kit (SQK-RNA002 from Oxford Nanopore Technology following the manufacturer’s instructions. The protocol was slightly modified: SuperScript IV reverse transcriptase (Invitrogen) was used instead of SuperScript III. The samples were incubated in a thermocycler at 50 °C for 35 min instead of 50 min, followed by inactivation at 70 °C for 15 min instead of 10 min before bringing the samples to 4 °C. DRS was then done on the flowcell FLO-MIN106 using Oxford Nanopore Technology (ONT) MinION sequencer according to the manufacturer’s protocol.

### Short-read sequencing using BGI technology

Total RNA extracted following the protocol described in the previous section was outsourced to BGI Japan. Total RNA quality was assessed by Bioanalyzer (Agilent) following the manufacturer’s protocol. After mRNA enrichment by oligo dT selection, the mRNA was fragmented and reverse transcribed. The second strand was synthesized, and the ends were repaired with A-tailing, resulting in double-stranded cDNA. Bubble adapters were then ligated. Fragments were amplified using PCR, heat-denatured into single strands and circularized. DNA nanoballs were synthesized and sequenced on a DNBSEQ-G400 platform.

### Cap Analysis of Gene Expression sequencing (CAGE-Seq)

Transcription start sites were defined using CAGE sequencing. CAGE library preparation, sequencing, mapping and quality filtering were outsourced to DNAFORM (Yokohama, Kanagawa, Japan). Total RNA extracted following protocol described in 2.3 was sent to DNAFORM. Total RNA quality was assessed by Bioanalyzer (Agilent) following the maker’s protocol. cDNAs were synthesized from total RNA using random primers. Ribose diols in the 5′ cap structures of RNA were oxidized, then biotinylated. The biotinylated RNA/cDNAs were selected by streptavidin beads (cap-trapping). After RNA digestion by RNaseONE/H and adaptor ligation to both ends of cDNA, double-stranded cDNA libraries (CAGE libraries) were constructed.

CAGE libraries were sequenced using single end reads of 75 nucleotides on a NextSeq 500 instrument (Illumina). The obtained reads (CAGE tags) were mapped to the genome of *S. pombe* 972h- from PomBase using BWA (v 0.5.9).^65^ Unmapped reads were then mapped by HISAT2 (v 2.0.5).^66^

### Transcriptome reannotation

Long reads from Nanopore sequencing was base called with ONT Guppy GPU Basecaller (v5.0.7). The quality of the reads were controlled with pycoQC (v 2.5.2).^67^ The reads were aligned to the reference genome of *S. pombe* 972h- using minimap2 (v 2.17).^68^ Reads were then converted to SAM and BAM files with the Samtools suite (v 1.14).^69^

Raw reads quality for short reads was assessed using FastQC v 0.11.9. The reads were then aligned to the genome of *S. pombe* 972h- using STAR aligner (v 2.7.9).^70^

Mapped CAGE reads obtained from the company were converted from bam to bed using the BEDTools suite (v 2.30.0).^71^ They were then analyzed using the CAGEfightR Bioconductor package (v 1.14.0).^72^

The transcriptome was reannotated using FLAIR (v 1.5),^73^ with a combination of the long (Nanopore) and short reads (BGI) described above. The annotation obtained by FLAIR was then corrected using SQANTI3 (v 4.2),^32^ by providing CAGE tags to validate the position of the 5′ ends. Gene and transcripts were then renamed using IsoAnnot Lite from tappAS pipeline (v 1.0.7).^74^ Using the default filter, a transcript is described as an artifact if “in the 20 bp downstream the annotated Transcription Terminating Site (TTS) there are 12 or more adenines at the genomic level,” “3′-end is an intrapriming artifact,” “has a junction that is labeled as RT-Switching,” “all junctions are either noncanonical or does not have a short read coverage above [the given] threshold” (citation from the SQANTI user manual).^32,33^

### Transcriptome analysis

Coverage depth and breadth for short reads was assessed using salmon (v 1.6.0) with the new transcriptome.^75^ For long reads, it was estimated using NanoCount (v 1.1.0).^76^ Counts from NanoCounts were inputted into the DESeq2 package (v 1.34.0) for differential gene expression analysis.^77^ Heatmaps were obtained using the ggplot2 package (v 3.3.5) for R.^78^ Gene ontology enrichment analysis was performed using clusterProfiler (v 3.10.1),^79^ enrichplot (v 1.14.1), DOSE (v 2.4.0) and ShinyGO (v 0.77).^80,81^

Alternative transcript isoforms were NanoCount quantification files as input. First, isoform switches are identified and ORFs are analyzed. From the resulting amino acid sequences, protein domains, coding potential, signal peptides and disordered regions were determined with CPC2,^82^ IUPred2A,^83^ hmmscan,^84^ and SignalP.^85^ Poly(A) tail length was estimated using nanopolish (v 0.14.0).^29^ Wilcoxon signed rank test was calculated to compare poly(A) tail lengths with scipy package (v 1.10.1) with the alternative hypothesis being “less.”^86^

### Calculation of the correlation score between sense and antisense gene expression

The compositional variance is described as a “cumulative measure of how far the number in a time series spread from the averages of the part they belong in a composition,” while the compositional covariance is a “measure of how changes in the part averages of a time series are associated with changes in the part averages of a second time series.”^87^ Combining the equations for both, the compositional correlation score r_c_ is defined by Equation (1):

(1) $$r_{c}=\frac{\sum_{i=1}^{k}\;\sum_{j=1}^{n_{i}}\;(A_{i,j}\text{-}\;\bar{A_{i}})(B_{i,j}-\bar{B_{i}})}{\sqrt{\left[\sum_{i=1}^{k}\;\sum_{j=1}^{n_{i}}\;{\left(A_{i,j}-\bar{A_{i}}\right)}^{2}][\sum_{i=1}^{k}\;\sum_{j=1}^{n_{i}}\;{\left(B_{i,j}-\bar{B_{i}}\right)}^{2}\right]}}$$

With:

*A*: scalar vector (first time series)

*B*: scalar vector (second time series)

*n*: the number of data in A

*k*: the number of parts in the current composition for which the correlation is being calculated

*n_i_*: the number of data in part *i*

*A_i,j_*: the *j*th data (time point) in *i*th part of vector *A*

*Ai*: the arithmetic mean of the *i*th part of vector *A*

Since in our data, we did not divide the time series in multiple parts *i*, the equation was simplified to the following Equation (2):

(2) $$r_{c}=\frac{\sum_{j=1}^{n_{i}}\;(A_{j}-\;\bar{A})(B_{i}-\bar{B})}{\sqrt{\left[\sum_{j=1}^{n_{i}}\;{\left(A_{j}-\bar{A}\right)}^{2}][\sum_{j=1}^{n_{i}}\;{\left(B_{i}-\bar{B}\right)}^{2}\right]}}$$

### Protein extraction and analysis

Cell pellets were sampled in the exact same way as for RNA extraction and protein was extracted from the cell pellets as following. Cells were grinded in liquid nitrogen on a pre-frozen mortar and a pestle. Lysis buffer (sodium deoxycholate (12 mM) (Wako), sodium N-dodecanoyl sarcosinate (Wako) (12 mM), ammonium bicarbonate (50 mM) (Nacalai Tesque), protease inhibitor cocktail (25X) (Roche)) was then added to the cells. After five rounds of cell grinding, the powder was transferred to a sterile Falcon tube. After thawing the cells on ice, they were transferred to a 1.5 mL sterile Eppendorf tube. The cells were then sonicated using Bioruptor II UCD-200 (20 min with intervals of 1 min). They were then centrifuged at 2,300 × *g*, 4 °C, for 10 min. After transferring the supernatants to a new 1.5 mL sterile Eppendorf tube, these were again centrifuged at 13,000 × *g*, 4 °C, for 10 min to further remove debris. Protein quantification was assessed using BCA protein assay kit from ThermoScientific following the manufacturer’s instructions. After adjusting the concentration to 1 mg/mL, 1 M of dithiothreitol was added to break down protein disulfide bonds. 1 M of iodoacetamide was added for peptide mapping purposes. After a 5-fold dilution in 50 mM ammonium bicarbonate, the proteins were digested by incubating the samples with 1 µg of Lys-C (Wako) at 37 °C for 3 h, then with 1 µg of trypsin from porcine pancreas (Sigma) at 37 °C for 16 h. After digestion, the sample was acidified with 1% trifluoroacetic acid (TFA) and sonicated for 10 min at intervals of 1 min. The supernatant was then desalted in C18-StageTips, dried under reduced pressure, and stored at –80 °C until analysis. The sample was dissolved in 0.1% formic acid 2% ACN and subjected to shotgun proteomic analysis using a nanoElute/timsTOFPro system (Bruker) equipped with a self-packed capillary column (ACQUITY UPLC BEH C18, Waters, 1.7 µm, 75 µm i.d., 250 mm length). The acquired mass spectra were analyzed as described in proteome analysis part.

### Proteome analysis

Proteomics analysis was carried out as follows. After measuring the mass of the peptides and proteins in our sample, MSFragger (v 3.4) handled peptide identification,^88^ and peptide-spectrum validation was done using PeptideProphet and ProteinProphet from Philosopher (v 3.4.13 and v 4.1.1).^89^ Label-free quantification with match between runs was done using IonQuant (v 1.5.5 and v 1.7.17) through the FragPipe pipeline v 16.0 (for mapping the proteins to database from UniProtKB) and v 17.1 (for mapping the proteins to database from the new resulting annotation).^90,91^ The database used for protein identification was downloaded from UniProtKB (accessed with 2021-09 release) with adding contaminants and false positives.^92^ Downstream analysis was performed using Bioconductor package MS-DAP v 1.0.^93^

## Supplementary Material

Supplemental Material

## DATA AVAILIBITY

The data that supports the findings of this study is deposited in Sequence Read Archive (SRA) database under the BioProject PRJNA990323 (shotgun cDNA-sequencing, long-read sequencing and CAGE-sequencing data) and in MassIVE under ProteomeXchange dataset PXD043446. The Supplementary Tables were made available at DOI: 10.17632/ssppwy9mdd.1, as well as a text file corresponding to our *S. pombe* reannotation “PomBaseplus” in GTF format.
