# Supplementary material for: Functional Consequences of Shifting Transcript Boundaries in Glucose Starvation
Source: Mol Cell Biol. 2023 Nov 17;43(11):611–28. doi: 10.1080/10985549.2023.2270406 (PMC10761120; doi:10.1080/10985549.2023.2270406)
Supplement: Supplemental Material [file TMCB_A_2270406_SM5891.zip › Supplementary Figure Captions.docx]

**Supplementary Figure 1**. Correlation between RNA expression at each time point

**Supplementary Figure 2**. Correlation between protein intensity at each time point

**Supplementary Figure 3**. Correlation between RNA expression and protein intensity at each time point
