# Supplementary figures and images for: Functional Consequences of Shifting Transcript Boundaries in Glucose Starvation
Source: Mol Cell Biol. 2023 Nov 17;43(11):611–28. doi: 10.1080/10985549.2023.2270406 (PMC10761120; doi:10.1080/10985549.2023.2270406)

# Supplementary Figure 1

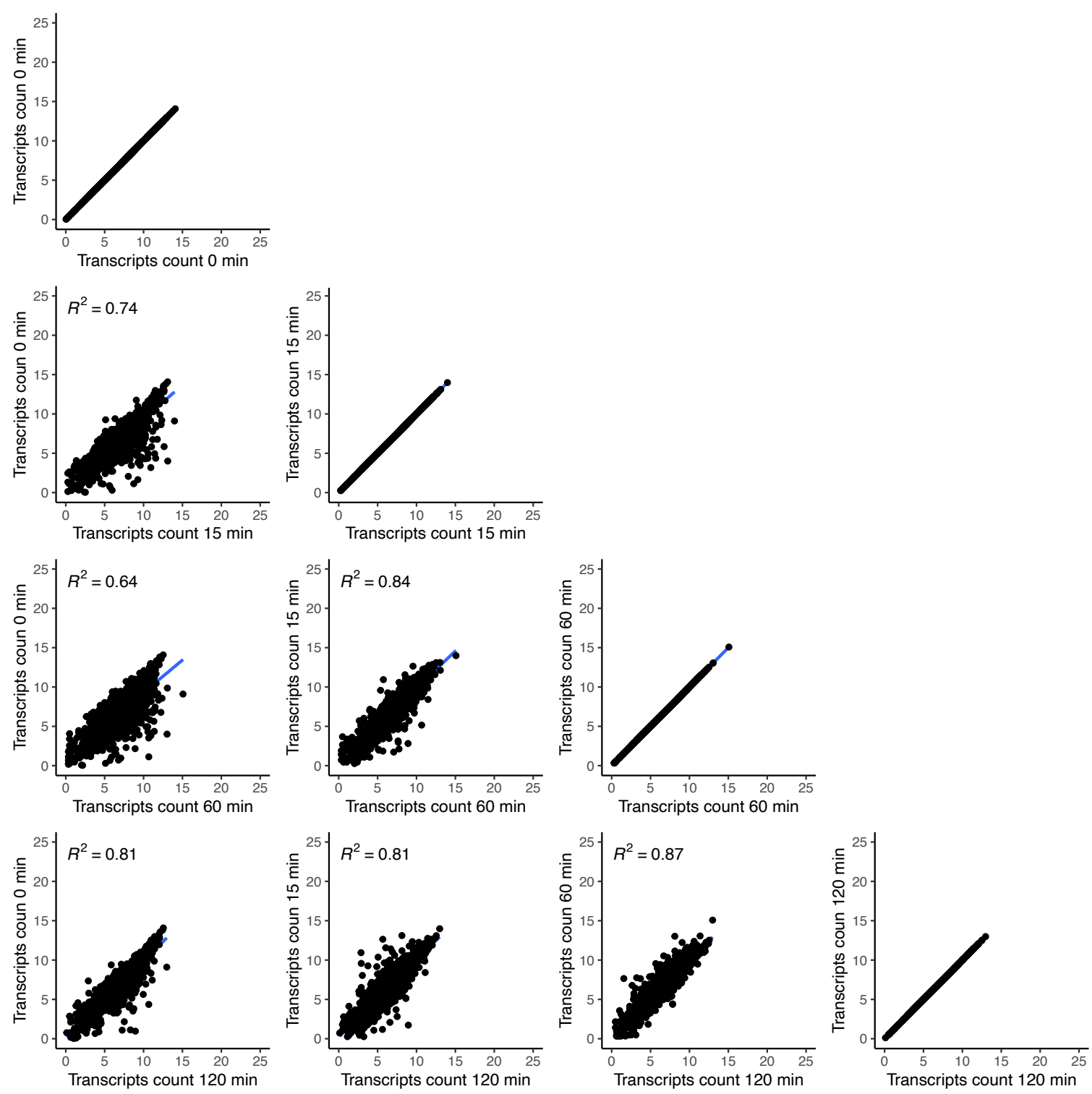

# Supplementary Figure 2

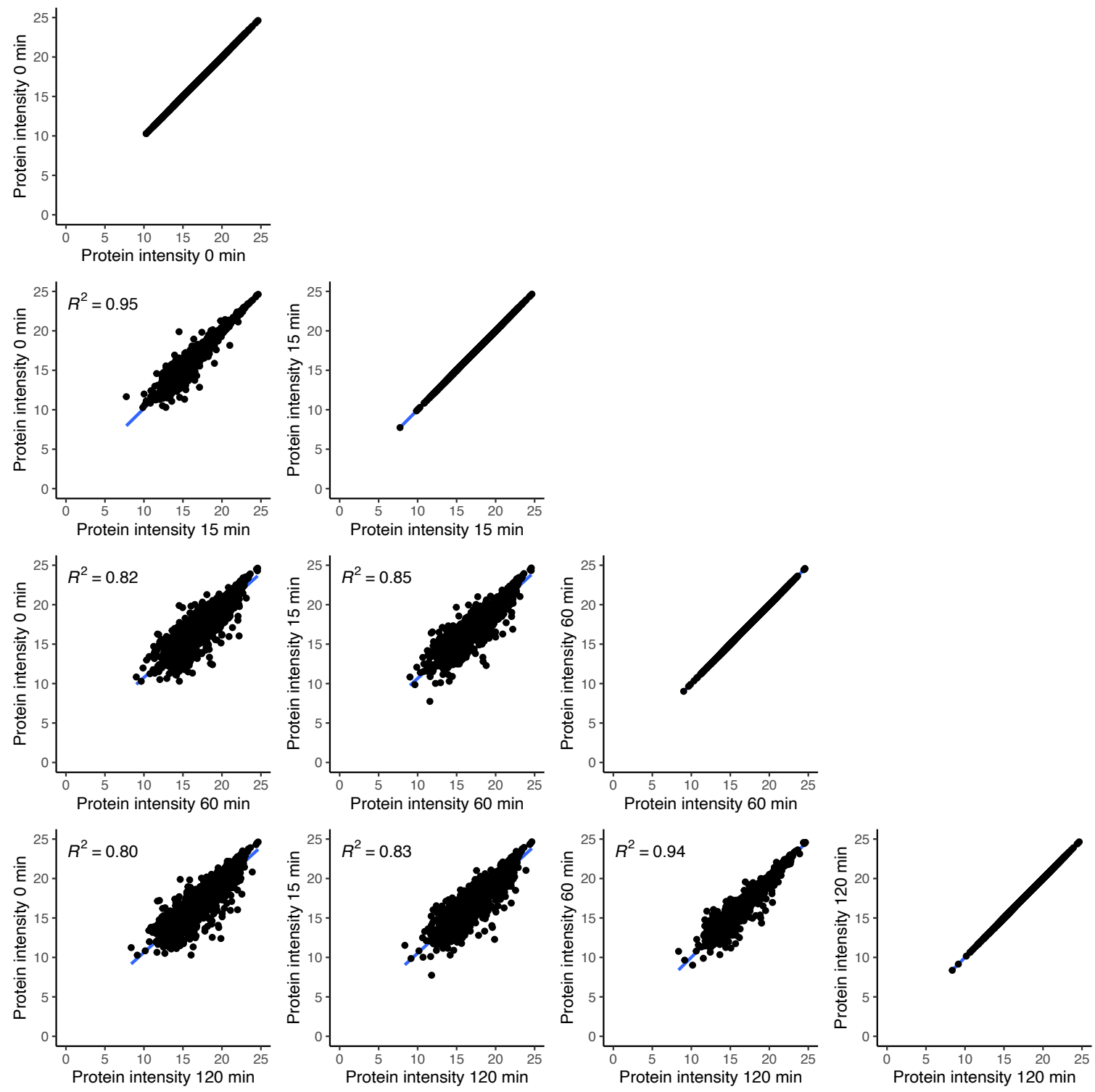

# Supplementary Figure 3

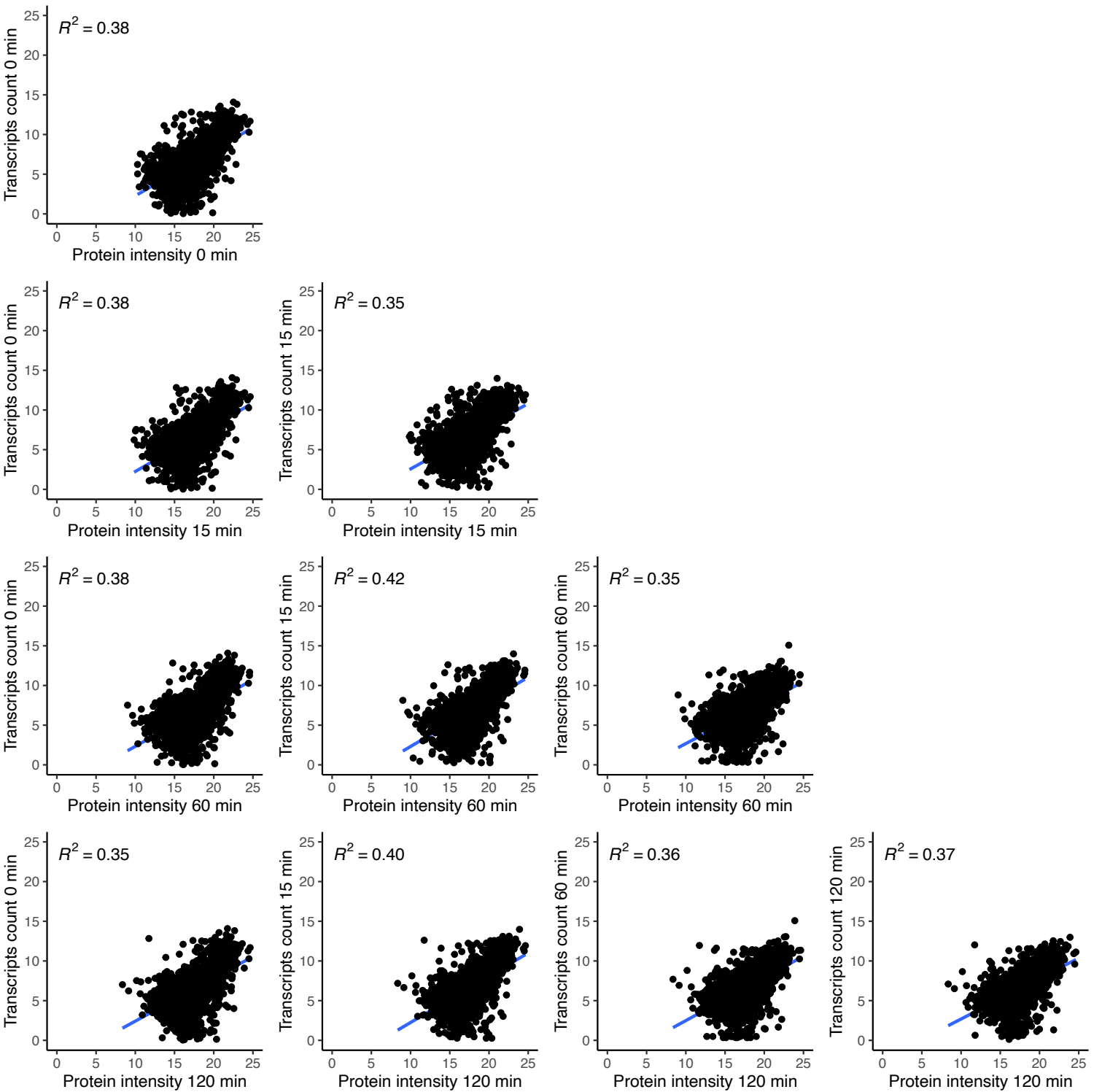

Supplement: Supplemental Material [file TMCB_A_2270406_SM5891.zip › Supplementary_Figures.pdf]
